# The draft genome sequence of “*Nitrospira lenta*” strain BS10, a nitrite oxidizing bacterium isolated from activated sludge

**DOI:** 10.1186/s40793-018-0338-7

**Published:** 2018-11-22

**Authors:** Dimitra Sakoula, Boris Nowka, Eva Spieck, Holger Daims, Sebastian Lücker

**Affiliations:** 1 Department of Microbiology, IWWR, Radboud University, Heyendaalseweg 135, 6525 AJ Nijmegen, Netherlands; 2 Department of Microbiology & Biotechnology, University of Hamburg, Ohnhorststr. 18, 22609 Hamburg, Germany; 3 Division of Microbial Ecology, Department of Microbiology and Ecosystem Science, University of Vienna, Althanstr. 14, 1090 Vienna, Austria

**Keywords:** “*Nitrospira lenta*”, Nitrite oxidation, *Nitrospira*, Wastewater treatment

## Abstract

The genus *Nitrospira* is considered to be the most widespread and abundant group of nitrite-oxidizing bacteria in many natural and man-made ecosystems. However, the ecophysiological versatility within this phylogenetic group remains highly understudied, mainly due to the lack of pure cultures and genomic data. To further expand our understanding of this biotechnologically important genus, we analyzed the high quality draft genome of “*Nitrospira lenta*” strain BS10, a sublineage II *Nitrospira* that was isolated from a municipal wastewater treatment plant in Hamburg, Germany. The genome of “*N. lenta*” has a size of 3,756,190 bp and contains 3968 genomic objects, of which 3907 are predicted protein-coding sequences. Thorough genome annotation allowed the reconstruction of the “*N. lenta*” core metabolism for energy conservation and carbon fixation. Comparative analyses indicated that most metabolic features are shared with *N. moscoviensis* and “*N. defluvii*”, despite their ecological niche differentiation and phylogenetic distance. In conclusion, the genome of “*N. lenta*” provides important insights into the genomic diversity of the genus *Nitrospira* and provides a foundation for future comparative genomic studies that will generate a better understanding of the nitrification process.

## Introduction

Nitrification, the two-step oxidation of ammonia to nitrate via nitrite, is a key process of the biogeochemical nitrogen cycle. Nitrite-oxidizing bacteria (NOB) are chemolithoautotrophic microorganisms that catalyze the oxidation of nitrite to nitrate, the second step of the nitrification process. For decades NOB where considered as metabolically restricted microorganisms solely associated with nitrification. However, experimental findings contradict this opinion, indicating a versatile ecophysiology of many NOB [1–4] and highlighting their important role in and possibly outside of the nitrogen cycle [5].

The genus *Nitrospira* is the most diverse known NOB genus and is divided in six different phylogenetic sublineages [6–8]. Members of the genus are ubiquitously present in different natural and engineered ecosystems [5, 9–11]. Despite their high abundance, only eleven representative species, distributed within the six *Nitrospira* sublineages, have been obtained in enrichment or pure culture so far [7, 8, 12–15]. Sublineage I and II *Nitrospira* are considered to be the dominant NOB in most wastewater treatment plants (WWTPs), playing a key role in the efficient removal of nitrogen via nitrification [6, 16]. Besides their widespread distribution and crucial role, the physiology of *Nitrospira* species is highly understudied, mainly due to the lack of pure cultures and genomic data [3, 17, 18]. The recent identification of complete ammonia-oxidizing (comammox) *Nitrospira* [15, 19] not only redefined the nitrification process, but also further indicated the importance of the genus and emphasized our poor understanding of the metabolic versatility present within this phylogenetic group.

“*Nitrospira lenta*” strain BS10 was isolated from a municipal WWTP [13] and it is the fourth isolate belonging to the sublineage II *Nitrospira*, besides *N. moscoviensis* [20], “*N. japonica*” [14], and the comammox organism “*N. inopinata*” [12]. Thus, insights into the “*N. lenta”* genome will shed light onto the genomic flexibility and metabolic diversity of the genus *Nitrospira* and aid further comparative studies between *Nitrospira* species.

## Organism information

### Classification and features

**Fig. 1:**
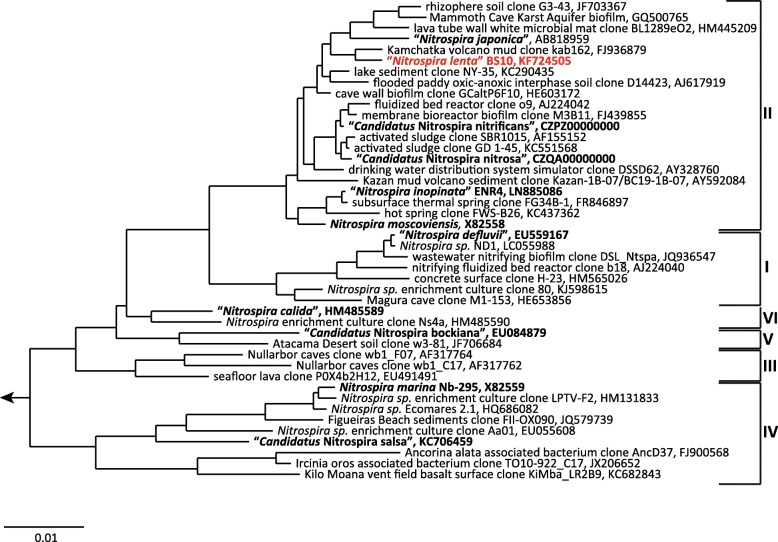
Phylogenetic analysis of selected representatives from the genus *Nitrospira*. A 16S rRNA gene-based maximum likelihood tree is shown. Sequences of cultured representatives are printed in bold, “*N. lenta*” in red. The tree was constructed using sequences ≥1165 bp and a 50% conservation filter, resulting in 1463 valid alignment positions. *Leptospirillum ferriphilum* (AF356829) and *L. ferrooxidans* (X86776) were used as outgroup, which is indicated by the arrow. Scale bar indicates 1% estimated sequence divergence

“*N. lenta*” strain BS10 is a Gram negative, aerobic NOB isolated from activated sludge of a municipal WWTP in Hamburg, Germany (basic properties are summarized in Table 1) [13]. Based on 16S rRNA gene-based phylogentic analysis, “*N. lenta*” is affiliated with *Nitrospira* sublineage II but is only distantly related to the sublineage II type strain, *N. moscoviensis* (Fig. 1).

**Table 1:** Classification and general features of “Nitrospira *lenta*” strain BS10 [34]

MIGS ID	Property	Term	Evidence code^a^
	Classification	Domain Bacteria	TAS [35]
		Phylum *Nitrospirae*	TAS [36]
		Class “*Nitrospira*”	TAS [36]
		Order “*Nitrospirales*”	TAS [36]
		Family “*Nitrospiraceae*”	TAS [36]
		Genus *Nitrospira*	TAS [32]
		Species *Nitrospira lenta*	TAS [13]
		Strain: BS10	TAS [13]
	Gram stain	Negative	TAS [13]
	Cell shape	Spiral-shaped rods	TAS [13]
	Motility	Non-motile^b^	TAS [13]
	Sporulation	Not reported	NAS
	Temperature range	10–32 °C	TAS [13]
	Optimum temperature	28 °C	TAS [13]
	pH range; Optimum	7.4–8.0; 7.4–7.6	TAS [13]
	Carbon source	Carbon dioxide	TAS [13]
MIGS-6	Habitat	Wastewater treatment plant	TAS [13]
MIGS-6.3	Salinity	0,5% *w*/*v*	TAS [13]
MIGS-22	Oxygen requirement	Aerobic	TAS [13]
MIGS-15	Biotic relationship	Free-living	TAS [13]
MIGS-14	Pathogenicity	Non-pathogen	NAS
MIGS-4	Geographic location	Germany/Hamburg	TAS [13]
MIGS-5	Sample collection	12/12/2006	TAS [13]
MIGS-4.1	Latitude	53° 31′ 8″ N	TAS [13]
MIGS-4.2	Longitude	9° 54′ 53″E	TAS [13]
MIGS-4.4	Altitude	–	NAS

^a^Evidence codes – *IDA* Inferred from Direct Assay, *TAS* Traceable Author Statement (i.e., a direct report exists in the literature), *NAS* Non-traceable Author Statement (i.e., not directly observed for the living, isolated sample, but based on a generally accepted property for the species, or anecdotal evidence). These evidence codes are from the Gene Ontology project [37]

^b^Genes encoding for a flagellum were identified in “*N. lenta*” BS10 genome

**Fig. 2:**
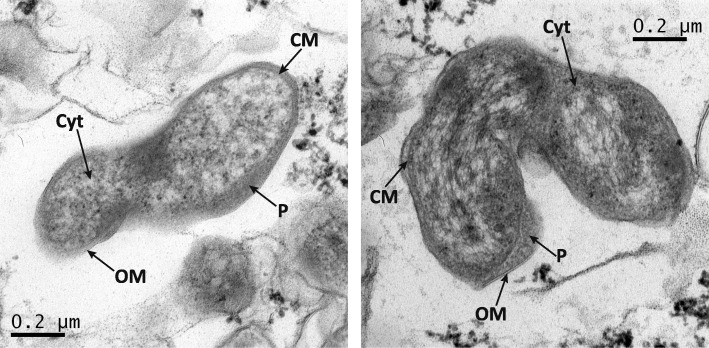
Electron micrographs of “*N. lenta*” strain BS10. The cells, in accordance to most of the bacteria belonging to the genus *Nitrospira*, feature a characteristic spiral shape and an enlarged periplasm. The individual cell components are indicated. OM, outer membrane; P, periplasm; CM, cytoplasmic membrane; Cyt, cytoplasm

A pure culture of “*N. lenta*” was obtained after applying a combination of standard isolation methods (density gradient centrifugation and serial dilutions) with an optical tweezer system for the sorting of single cells [13]. “*N. lenta*” grows mainly planktonic and forms helical-shaped cells. Cells are 1.0–2.3 μm in length and 0.2–0.3 μm in diameter (Table 1, Fig. 2). As shown by Nowka et al. [13], “*N. lenta*” is able to grow at lower temperatures (10 °C) than *N. moscoviensis*. Interestingly, while “*N. lenta*” is not able to tolerate high concentrations of nitrite, it exhibits a lower affinity for nitrite compared to *N. moscoviensis* and “*N. defluvii*”, which indicates a clear niche differentiation among these *Nitrospira* species [21].

## Genome sequencing information

### Genome project history

“*N. lenta*” was selected for whole genome sequencing on the basis of its relevance within the nitrogen cycle as well as due to the general lack of genomic information for *Nitrospira* species. Furthermore, because “*N. lenta*” was isolated from activated sludge, its genome was expected to yield insights that allow optimization and stabilization of the nitrification process in wastewater treatment. The draft genome sequence of “*N. lenta*” BS10 was completed on 27/07/2013. The high-quality draft genome is available in the European Nucleotide Archive (ENA) under study accession number PRJEB26290. An overview of the genome sequencing project is given in Table 2.

**Table 2:** Project information

MIGS ID	Property	Term
MIGS 31	Finishing quality	High quality draft
MIGS-28	Libraries used	1
MIGS 29	Sequencing platforms	Roche GS FLX
MIGS 31.2	Fold coverage	40
MIGS 30	Assemblers	GS De Novo Assembler
MIGS 32	Gene calling method	AMIGene [24]
	Locus Tag	NITLEN
	GenBank ID	OUNR00000000.1
	GenBankDate of Release	4 June 2018
	GOLD ID	–
	BioProject	PRJEB26290
MIGS 13	Source Material Identifier	BS10
	Project relevance	Microbiology, Biotechnology

### Growth conditions and genomic DNA extraction

“*N. lenta*” was cultivated as described by Nowka et al. [13] in mineral salt medium amended with 0.02 g L^− 1^ NaNO_2_^−^ as energy source. The cultures were incubated in the dark at 28 °C, with moderate stirring (100–300 rpm). The genomic DNA was extracted following the hexadecyltrimethylammoniumbromide (CTAB) protocol provided by the DOE Joint Genome Institute (JGI, https://jgi.doe.gov/user-program-info/pmo-overview/protocols-sample-preparation-information/) as described elsewhere [22].

### Genome sequencing and assembly

High-throughput sequencing was performed at GATC Biotech (Constance, Germany) using Roche GS FLX technology. The final draft genome of “*N. lenta*” was obtained using the GS De Novo Assembler (Newbler) and comprised 3.8 Mb on 22 contigs. Genome completeness was evaluated with CheckM [23]. Similarly to the complete genomes of “*N. defluvii*” (98% completeness, 2.3% contamination) and *N. moscoviensis* (96% completeness, 6.6% contamination), the “*N. lenta*” genome was estimated to be 96% complete with 3.2% contamination.

### Genome annotation

The draft genome of “*N. lenta*” was annotated using the MicroScope platform [24] as described in detail elsewhere [17]. The automatic annotation was manually checked and curated using the MicroScope Web interface MaGe [25]. The genomic features of “*N. lenta*” were compared to *N. moscoviensis* and “*N. defluvii*”, the type strains of the *Nitrospira* sublineages II and I, respectively, using the OrthoVenn web service [26] for the identification and comparison of orthologous gene groups. Sequence similarities were calculated using an E-value of 1e-05. An inflation value of 1.5 was applied to generate the orthologous clusters.

## Genome properties

**Fig. 3:**
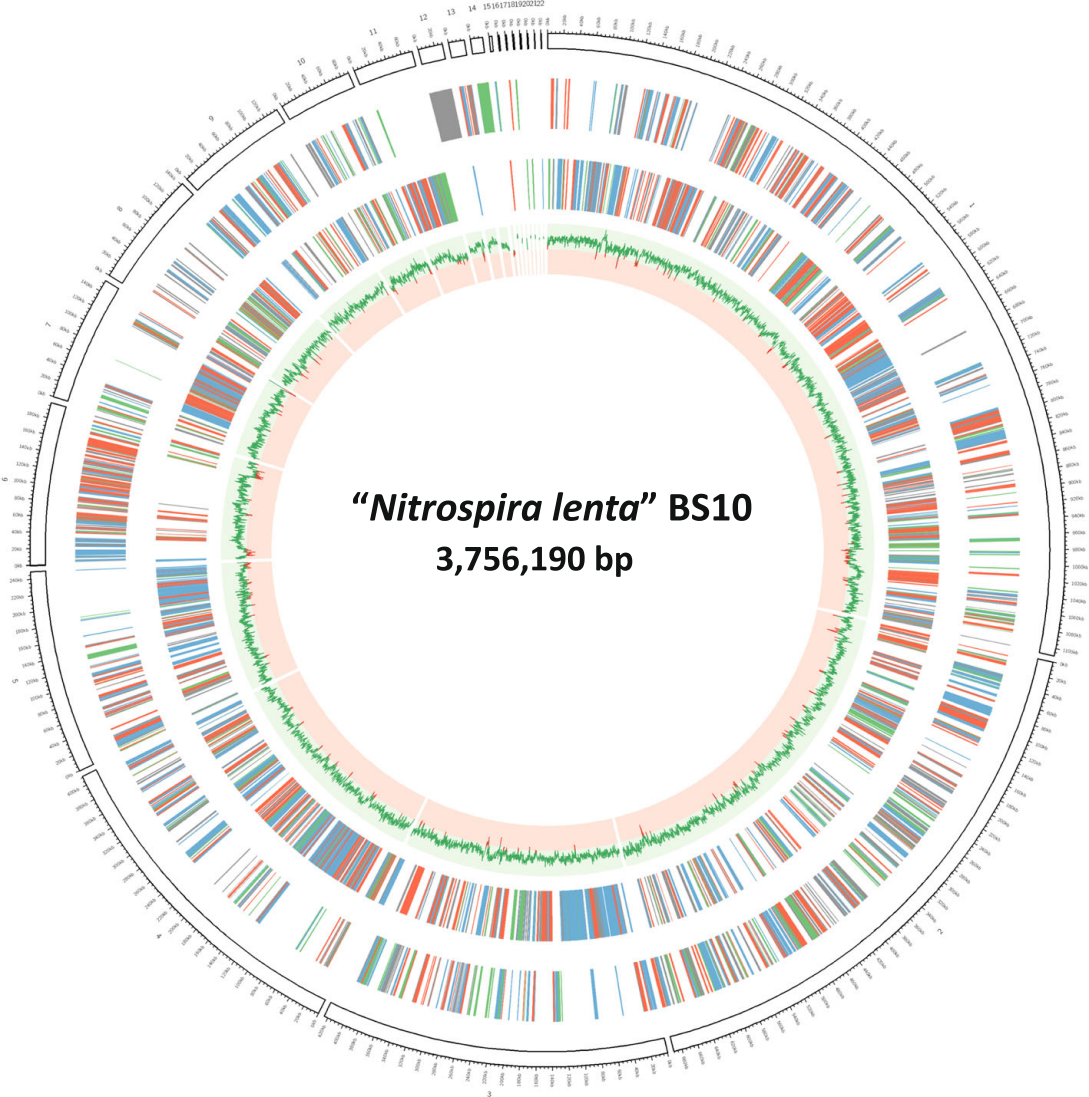
Circular representation of the “*N. lenta*” genome. From inside out the circles display: (1) G + C content (red < 50%, green > 50%), (2) CDS on reverse strand, (3) CDS on forward strand, (4) contig organization. The tick marks correspond to 20 kbp. CDS colors indicate COG classification (blue, cellular processes and signaling; green, information storage and processing; red, metabolism, grey; poorly characterized). Contigs are ordered by size, and their arrangement in the figure may not reflect the positions of the respective regions on the chromosome. The graphical circular map of “*N. lenta*” chromosome was generated using the CIRCOS software [33]

The “*N. lenta*” draft genome consists of 22 contigs and has a total size of 3,756,190 bp with an overall G + C content of 57.9% (Fig. 3, Table 3). From a total of 3968 predicted genes, 3907 (98.5%) and 61 (1.5%) are protein and RNA coding sequences, respectively. The genome encodes for 1 complete rRNA operon and 46 tRNAs, with 1 to 5 copies for each tRNA type. Moreover, 66.8% of the predicted genes were assigned into to Clusters of Orthologous Groups (COG) functional categories (Table 4).

**Table 3:** Genome statistics

Attribute	Value
Genome size (bp)	3,756,190
DNA coding (bp)	3,487,097
DNA G + C (%)	57.9
DNA scaffolds	22
Total genes	3968
Protein coding genes	3907
RNA genes	61
rRNA genes	3
tRNA genes	46
Pseudo genes	0
Genes in internal clusters	–
Genes with function prediction	1990
Genes assigned to COGs	2609
Genes with Pfam domains	3868
Genes with signal peptides^a^	196
Genes with transmembrane helices	965
CRISPR repeats	0

^a^Only signal peptides with a prediction probability greater 70% were taken into consideration

**Table 4:** Number of genes associated with general COG functional category prediction

Code	Value	%age^a^	Description
J	163	4.17	Translation, ribosomal structure and biogenesis
A	0	0	RNA processing and modification
K	148	3.79	Transcription
L	142	3.64	Replication, recombination and repair
B	1	0.03	Chromatin structure and dynamics
D	48	1.23	Cell cycle control, Cell division, chromosome partitioning
V	44	1.13	Defense mechanisms
T	256	6.55	Signal transduction mechanisms
M	303	7.76	Cell wall/membrane biogenesis
N	114	2.92	Cell motility
U	119	3.05	Intracellular trafficking and secretion
O	151	3.87	Posttranslational modification, protein turnover, chaperones
C	205	5.25	Energy production and conversion
G	133	3.40	Carbohydrate transport and metabolism
E	198	5.07	Amino acid transport and metabolism
F	59	1.51	Nucleotide transport and metabolism
H	136	3.48	Coenzyme transport and metabolism
I	76	1.95	Lipid transport and metabolism
P	156	3.99	Inorganic ion transport and metabolism
Q	77	1.97	Secondary metabolites biosynthesis, transport and catabolism
R	389	9.96	General function prediction only
S	221	5.66	Function unknown
–	1298	33.22	Not in COGs

^a^The total is based on the total number of protein coding genes in the genome

## Insights from the genome sequence

**Fig. 4:**
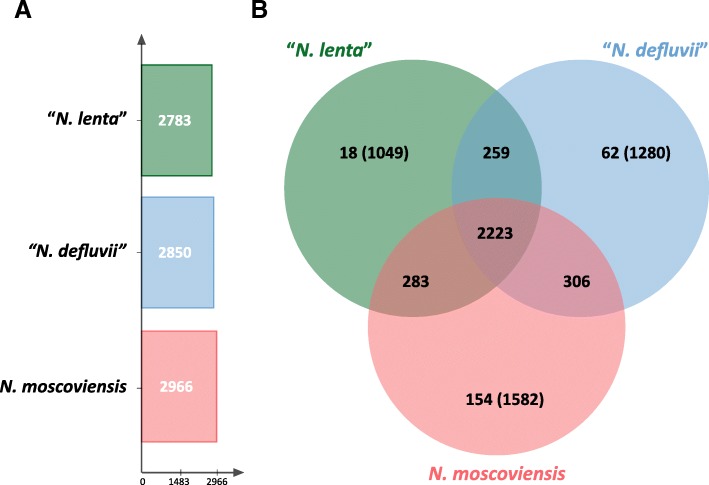
Genome comparison of “*N. lenta*” with representatives of *Nitrospira* sublineages I and II. (**a**) Total number of inferred orthologous protein clusters for each species. (**b**) Venn diagram depicting the distribution of orthologous clusters among the genomes of “*N. lenta*”, “*N. defluvii*” and *N. moscoviensis*. The numbers indicate orthologous protein clusters; numbers in brackets depict the unique, unclustered proteins of each genome

*Nitrospira* species belonging to sublineages I and II are the most abundant NOB in many environments and play a key role in N-cycling in engineered ecosystems [6, 10]. Recent experimental data indicates a clear niche differentiation between sublineage I and II *Nitrospira* [21, 27]. More specifically, “*N. lenta*”, like other members of sublineage II, exhibits a lower maximum activity [21, 27] and could be outcompeted by sublineage I *Nitrospira* at higher nitrite concentrations [28]. Despite their ecophysiological differences, sublineage I and II *Nitrospira* exhibit substantial genomic similarities. More specifically, “*N. lenta*” shares a core genome including 2223 orthologous protein clusters with *N. moscoviensis* and “*N. defluvii*”. This corresponds to 67.3% of the pan-genome of the *Nitrospira* species included in this analysis (Fig. 4). Moreover, “*N. lenta*” features the lowest number of unique genes (1100, of which 51 are grouped in 18 paralogous protein clusters). Most of these unique genes lack any function prediction (Fig. 4).

**Fig. 5:**
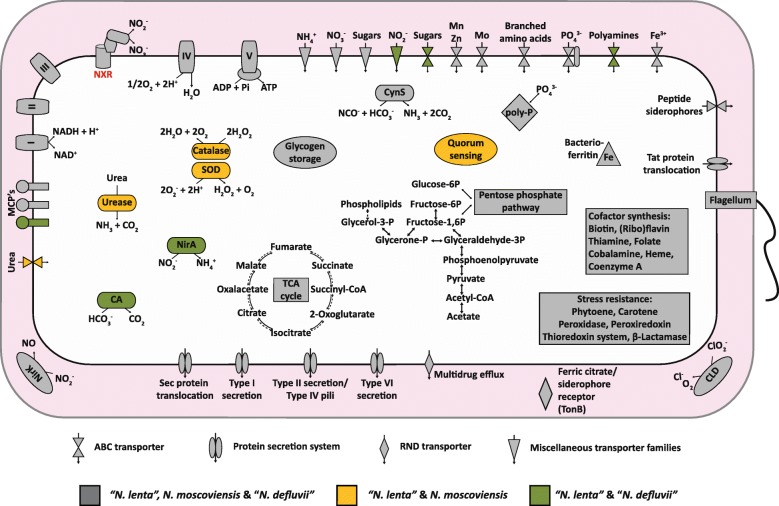
Cell metabolic cartoon of “*N. lenta*” in comparison to *N. moscoviensis* and “*N. defluvii*”. CA, carbonic anhydrase; SOD, superoxide dismutase; CLD, chlorite dismutase; CynS, cyanate hydratase (cyanase); MCPs, methyl-accepting chemotaxis proteins; NirA, ferredoxin nitrite reductase; NirK, dissimilatory nitrite reductase. Enzyme complexes of the electron transport chain are labeled by Roman numerals

In accordance with its ability to oxidize nitrite to nitrate [13], “*N. lenta*” encodes all proteins required for nitrite oxidation (Fig. 5), for which the key enzyme is a membrane-associated nitrite oxidoreductase (NXR) [29]. This protein complex belongs to the type II dimethyl sulfoxide reductase family of molybdopterin-binding enzymes and consists of three subunits [17, 29]. The “*N. lenta*” genome contains two paralogous copies of *nxrA* and *nxrB*, encoding the NXR α and β subunits, respectively, and two copies of *nxrC* for the candidate γ subunit. Like all *Nitrospira* genomes analyzed to date, the “*N. lenta*” genome contains *nirK*, encoding the copper-dependent NO-forming nitrite reductase. While the function of NirK in *Nitrospira* is still unclear, a role in dissimilatory nitrite reduction is unlikely as no other genes involved in denitrification were identified in “*N. lenta*” or any other *Nitrospira*. One cannot exclude the possibility that NO plays a regulatory role in *Nitrospira*, for example in the regulation of forward versus reverse electron flow as proposed for *Nitrobacter* [30]. Moreover, “*N. lenta*” exhibits the genetic capacity for nitrogen assimilation from nitrite as its genome features *nirA* encoding the ferredoxin-dependent nitrite reductase. Interestingly, NirA is conserved in “*N. defluvii*”, but not the other genome-sequenced sublineage II *Nitrospira*, which either encode an octaheme nitrite reductase [3, 18], or, in the case of the comammox *Nitrospira*, lack assimilatory nitrite reduction pathways [15, 19, 31]. Interestingly, the “*N. lenta*” BS10 genome also features an *ure* operon encoding a functional urease (UreC), as well as a complete gene set (urtABCDE) for a high affinity urea ABC transporter. This implies that “*N. lenta*” is able to hydrolyse urea to ammonium and CO_2_, facilitating nitrogen and carbon assimilation from urea and reciprocal feeding between “*N. lenta*” and urease-negative ammonia-oxidizing bacteria [3, 4]. The “*N. lenta*” urease is closely related to the enzyme of *N. moscoviensis*, but significantly differs from known ureases of ammonia-oxidizing bacteria [3].

“*N. lenta*” conserves energy by nitrite oxidation with oxygen as terminal electron acceptor. During nitrite oxidation catalyzed by NXR, two electrons are shuttled (putatively via cytochrome *c*) towards a putative novel *bd-*like terminal oxidase [17]. The proton motive force established through active proton pumping by this novel complex IV and/or the release of scalar protons during nitrite oxidation drives ATP synthesis by a F_O_F_1_-type ATPase (complex V). The other respiratory complexes (complexes I to III) will not contribute to energy conservation during lithoautotrophic growth on nitrite, but will operate in reverse to provide reducing equivalents for carbon fixation [17]. Moreover, the complete gene repertoire for the oxidative and reductive tricarboxylic acid (TCA) cycle is present in “*N. lenta*” for pyruvate oxidation via acetyl-CoA and CO_2_ fixation, respectively. Moreover, the complete glycolysis/gluconeogenesis and pentose phosphate pathways were identified. The observed presence of glycolysis and the oxidative TCA cycle might indicate that “*N. lenta*” can benefit from a mixotrophic lifestyle in the presence of organic carbon, as has been reported for other *Nitrospira* representatives [6, 16, 32].

Finally, the “*N. lenta*” genome contains various defense mechanisms against heavy metals, antibiotics, and the antibacterial agent acriflavine. “*N. lenta*” encodes a superoxide dismutase for defense mechanisms against oxidative stress, as well as several bacterioferritins, which can participate in oxidative stress resistance mechanisms [17].

## Conclusions

Together with *N. moscoviensis* and “*N. japonica*”, “*N. lenta*” represents only the third cultured species of canonical nitrite-oxidizing *Nitrospira* from sublineage II. In this study, the genome of “*N. lenta*” was analyzed, demonstrating that “*N. lenta*” shares a significant amount of genomic features with other representatives of the genus. However, physiological differences observed by Nowka et al. regarding growth conditions and nitrite affinities [13, 21] clearly suggest a niche differentiation between different species. The “*N. lenta*” genome will facilitate a better understanding of the metabolic versatility of the genus *Nitrospira* and will be useful for future comparative studies, especially those with a focus on species obtained from engineered systems.

## Abbreviations

NOB: Nitrite-oxidizing bacteria
NXR: Nitrite oxidoreductase
WWTP: Wastewater treatment plant
